# Supplementary material for: Predicting Heteropolymer Phase Separation Using Two-Chain Contact Maps
Source: ArXiv. 2025 May 20:arXiv:2503.04659v2. Originally published 2025 Mar 6. Preprint. [Version 2] (PMC11908358)
Supplement: Supplement 1 [file NIHPP2503.04659v2-supplement-1.pdf]

SUPPLEMENTAL INFORMATION

B_{22}-matched HP model datasets

The following tables provide the sequence information and ϵ values for HP copolymers at constant B_{22} values of $-400\sigma^3$ and $-1000\sigma^3$. The epsilon values were tuned to achieve the same homotypic B_{22} values (within statistical error) across four trials. Note that the sequences differ slightly between the tables, because sequences that did not exhibit clear phase separation or aggregation in our direct-coexistence simulations were excluded. The fraction of H monomers in each sequence is denoted by x_{H}. PS reports whether the sequence phase separates (✓ = yes, X = no). The complete dataset can be obtained at `https://github.com/wmjac/heteropolymer-phase-separation`

TABLE I: Sequence information for B_{22} = -400 σ^3

Index	Sequence	x_{H}	ϵ	PS
1	PHPHPPHPPHPPHPPHPHPH	0.4	2.285	X
2	HHPPPPHPPHHPHPHPHPPP	0.4	1.762	X
3	HPPHPPHPPHPPHPPHPHPH	0.4	2.222	X
4	PHPHPPHPPHPPHPPHPPHH	0.4	2.030	X
5	HPHPHPPHPHPPPPHPHPPH	0.4	2.137	X
6	PHPPPPPHHHHPHHPPPHPP	0.4	1.385	X
7	HPPPHHHPHPPHPPPHPPPH	0.4	1.700	X
8	PPHHHPHPHPPPPPHPHPPH	0.4	1.705	X
9	PPHPPPHPPHPHHHHHPPPP	0.4	1.317	X
10	PHHHPPHPPPHPHPPPPHPH	0.4	1.790	X
11	HPHPHPHPHPHPHPHPHPHP	0.5	1.770	X
12	HPPHPPHHPHPHHPHPHPHP	0.5	1.550	X
13	HPHHPPPHPPHHHPPPHPHH	0.5	1.344	X
14	PPPHHPHHHPHPPHPPHPHH	0.5	1.295	X
15	PPHHPPHPPHPHHPHPPHHH	0.5	1.320	X
16	PHPPPHHHPPHHHPHHHPPP	0.5	1.172	X
17	PPHHHPPHPPHPPHPPHHHH	0.5	1.216	X
18	HHHHPPHHPPPHPPHHPPHP	0.5	1.216	X
19	HHHHPPPHHHHPPPPPHPPH	0.5	1.068	X
20	HPHHPHHHHPHPHPPPPPHP	0.5	1.147	X
21	HHHPPPHHHPPHHHPPPHHH	0.6	0.970	✓
22	HPHPPPHHHHPHHHPHPHHP	0.6	1.019	X
23	PHPHPPHHPHPHHHHPPHHH	0.6	1.000	X
24	HHHHPPHHPHHHPHPPHPHP	0.6	0.988	X
25	HHHHPPPHHPHHPHHHHPPP	0.6	0.938	X
26	HHHHPPHPHHHPPHHHHPPP	0.6	0.937	X
27	HHHHPPHHHPPHHHHPPPPH	0.6	0.918	X
28	PHHPHPHHHHHHHPPPPHPH	0.6	0.910	X
29	HHHPPHHHHHPHHPPHPPHP	0.6	0.926	X
30	HPPPPHHPHHHHHHHPPHPH	0.6	0.884	X
31	PHPPPPHHHHHPHHPHPHHH	0.6	0.913	X
32	HHHHHHPPHHHPPHPPHPHP	0.6	0.886	X
33	HHHHPHHPHHPHPHHPHHHH	0.75	0.748	✓
34	HPHPHHHHHHHHHPHPHHHP	0.75	0.699	X
35	HHPHHPHHHHHHHHHPHHPP	0.75	0.672	X
36	PHPHHHHHHHHHHHHPHPPH	0.75	0.641	X
37	HHHHHHHHHHPHHHPHPHPP	0.75	0.638	X
38	PPHPHPHHPHHHHHHHHHHH	0.75	0.630	X
39	PPHPPHHHHPHHHHHHHHHH	0.75	0.622	X
40	PPHPHPHPHHHHHHHHHHHH	0.75	0.614	X
41	HHHHHHHHPPPPHHHHHHHH	0.8	0.580	✓
42	PPHHPHHHHHHHHHHHHHPH	0.8	0.586	X
43	PPHHHHHHHHHHHHHHHHPP	0.8	0.564	X
44	PPPHHPHHHHHHHHHHHHHH	0.8	0.555	X
45	PPPPHHHHHHHHHHHHHHHH	0.8	0.536	X
46	HHHHHHHHHHHHHHHHHHHH	1.0	0.420	✓
47	PHHHPHPHPHPPPHPPPHHH	0.5	1.300	X
48	PHPPPHHHHHPHPHPHHPHH	0.6	0.985	X
49	HHPHHHPHHHPPHPPHPHPH	0.6	1.020	X
50	HHPHHHHPHPPHHPHPPPHH	0.6	1.000	X
51	HPHHPHHHPPPHHPHPHPHH	0.6	1.110	✓
52	HHHHPPPHPHHPHHPHPHPH	0.6	1.053	X
53	HHPHPHHHPHPHPPPHHPHH	0.6	1.070	✓
54	HHHHHPPHPPPHHHPPPHHH	0.6	0.915	X
55	PHHHHPHHPHPHHPPHHPPH	0.6	1.070	X
56	PHPHHHHPHHPHPPPHHHHP	0.6	1.010	X
57	HPHHPPHPHPPHHHPPHHHH	0.6	1.010	X
58	HHPHHPHPPHPHHHPPHPHH	0.6	1.080	✓
59	HPHHHPHPHPHPPHHPHHHP	0.6	1.120	X
60	HPHHPHHPPHHPPHHPHHPH	0.6	1.150	✓
61	HPHHPHPHPHHPHPHPHHPH	0.6	1.200	✓
62	HPHHPHHPHPHPHHPHHPHP	0.6	1.180	X
63	HHHPHHPHHHPHHPHHPHHH	0.75	0.766	✓
64	PHHHPHHHPHHHPHHHPHHH	0.75	0.750	✓
65	HHHPHPHHHPPHHPHHHHHH	0.75	0.720	✓
66	HHPHHHHHHPHPHHPHHHHP	0.75	0.735	✓
67	HHHHHPHPHHPHHHPHHHPH	0.75	0.738	✓
68	HHHPHPHHHHHHPHHHPHHP	0.75	0.720	✓
69	HPHHHPHPHHHPHHHPHHHH	0.75	0.750	✓
70	HHPHPHHHHPHHHHHHHHHP	0.8	0.630	✓
71	HHPHHHHHHPHHHHHPHHPH	0.8	0.650	✓
72	HHHHHPHHHPHHHHPHPHHH	0.8	0.650	✓
73	HPHHPPHHHHHHHHHHPHHH	0.8	0.620	✓
74	HPPHHHHHPHHHHHHHPHHH	0.8	0.628	✓
75	HHHPHHHPHHHHPHHHPHHH	0.8	0.670	✓

Continued on next page

TABLE II: Sequence information for B_{22} = -1000 σ^3

Index	Sequence	x_{H}	ϵ	PS
1	PHPHPPHPPHPPHPPHPHPH	0.4	2.442	X
2	HHPPPPHPPHHPHPHPHPPP	0.4	1.871	X
3	HPPHPPHPPHPPHPPHPHPH	0.4	2.385	X
4	PHPHPPHPPHPPHPPHPPHH	0.4	2.166	X
5	HPHPHPPHPHPPPPHPHPPH	0.4	2.296	X
6	PHPPPPPHHHHPHHPPPHPP	0.4	1.506	X
7	HPPPHHHPHPPHPPPHPPPH	0.4	1.830	X
8	PPHHHPHPHPPPPPHPHPPH	0.4	1.840	X
9	PPHPPPHPPHPHHHHHPPPP	0.4	1.438	X
10	PHHHPPHPPPHPHPPPPHPH	0.4	1.920	X
11	HPHPHPHPHPHPHPHPHPHP	0.5	1.960	✓
12	HPPHPPHHPHPHHPHPHPHP	0.5	1.687	X
13	PPPHHPHHHPHPPHPPHPHH	0.5	1.407	X
14	PPHHPPHPPHPHHPHPPHHH	0.5	1.445	X
15	PHPPPHHHPPHHHPHHHPPP	0.5	1.270	X
16	PPHHHPPHPPHPPHPPHHHH	0.5	1.340	X
17	HHHHPPHHPPPHPPHHPPHP	0.5	1.335	X
18	HHHHPPPHHHHPPPPPHPPH	0.5	1.170	X
19	HPHHPHHHHPHPHPPPPPHP	0.5	1.260	X

Continued on next page

Index	Sequence	x_{H}	ϵ	PS
20	HHHPPPHHHPPHHHPPPHHH	0.6	1.085	✓
21	HPHPPPHHHHPHHHPHPHHP	0.6	1.125	X
22	PHPHPPHHPHPHHHHPPHHH	0.6	1.112	X
23	HHHHPPHHPHHHPHPPHPHP	0.6	1.100	X
24	HHHHPPPHHPHHPHHHHPPP	0.6	1.040	X
25	HHHHPPHPHHHPPHHHHPPP	0.6	1.037	X
26	HHHHPPHHHPPHHHHPPPPH	0.6	1.022	X
27	PHHPHPHHHHHHHPPPPHPH	0.6	1.010	X
28	HHHPPHHHHHPHHPPHPPHP	0.6	1.030	X
29	HPPPPHHPHHHHHHHPPHPH	0.6	0.990	X
30	PHPPPPHHHHHPHHPHPHHH	0.6	1.020	X
31	HHHHHHPPHHHPPHPPHPHP	0.6	1.010	X
32	HHHHPHHPHHPHPHHPHHHH	0.75	0.860	✓
33	HPHPHHHHHHHHHPHPHHHP	0.75	0.793	✓
34	HHPHHPHHHHHHHHHPHHPP	0.75	0.769	X
35	PHPHHHHHHHHHHHHPHPPH	0.75	0.735	X
36	HHHHHHHHHHPHHHPHPHPP	0.75	0.727	X
37	PPHPHPHHPHHHHHHHHHHH	0.75	0.720	X
38	PPHPPHHHHPHHHHHHHHHH	0.75	0.708	X
39	PPHPHPHPHHHHHHHHHHHH	0.75	0.703	X
40	HHHHHHHHPPPPHHHHHHHH	0.8	0.674	✓
41	PPHHPHHHHHHHHHHHHHPH	0.8	0.676	X
42	PPHHHHHHHHHHHHHHHHPP	0.8	0.644	X
43	PPPHHPHHHHHHHHHHHHHH	0.8	0.640	X
44	PPPPHHHHHHHHHHHHHHHH	0.8	0.614	X
45	HHHHHHHHHHHHHHHHHHHH	1.0	0.497	✓
46	PHPPPHHHHHPHPHPHHPHH	0.6	1.097	X
47	HHPHHHPHHHPPHPPHPHPH	0.6	1.143	X
48	HHPHHHHPHPPHHPHPPPHH	0.6	1.120	✓
49	HPHHPHHHPPPHHPHPHPHH	0.6	1.227	✓
50	HHHHPPPHPHHPHHPHPHPH	0.6	1.168	✓
51	HHPHPHHHPHPHPPPHHPHH	0.6	1.197	✓
52	HHHHHPPHPPPHHHPPPHHH	0.6	1.028	✓
53	PHPHHHHPHHPHPPPHHHHP	0.6	1.117	X
54	HHPHHPHPPHPHHHPPHPHH	0.6	1.218	✓
55	HPHHHPHPHPHPPHHPHHHP	0.6	1.247	✓
56	HPHHPHHPPHHPPHHPHHPH	0.6	1.303	✓
57	HPHHPHPHPHHPHPHPHHPH	0.6	1.368	✓
58	HPHHPHHPHPHPHHPHHPHP	0.6	1.326	✓
59	HHHPHHPHHHPHHPHHPHHH	0.75	0.880	✓
60	PHHHPHHHPHHHPHHHPHHH	0.75	0.865	✓
61	HHHPHPHHHPPHHPHHHHHH	0.75	0.818	✓
62	HHPHHHHHHPHPHHPHHHHP	0.75	0.835	✓
63	HHHHHPHPHHPHHHPHHHPH	0.75	0.850	✓
64	HHHPHPHHHHHHPHHHPHHP	0.75	0.822	✓
65	HPHPHHHHPHHHHHPHHHHP	0.75	0.822	✓
66	HPHHHPHPHHHPHHHPHHHH	0.75	0.857	✓
67	HHPHPHHHHPHHHHHHHHHP	0.8	0.729	✓
68	HHPHHHHHHPHHHHHPHHPH	0.8	0.747	✓
69	HHHHHPHHHPHHHHPHPHHH	0.8	0.758	✓
70	HPHHPPHHHHHHHHHHPHHH	0.8	0.710	✓
71	HPPHHHHHPHHHHHHHPHHH	0.8	0.720	✓
72	HHHPHHHPHHHHPHHHPHHH	0.8	0.776	✓

Curated HPS IDP model challenge dataset

The following table provides the sequence information and B_{22} values for HPS IDP heteropolymers that constitute the "challenge set" shown in Figure 6B. The complete dataset can be found in Ref. [41].

TABLE III: Sequence information for the curated HPS IDP model challenge dataset

Index	Sequence	B_{22} ($\AA^3$)	PS
1	RRRKKKFKWQTLWAEETEEEE	-93000	✓
2	KRTRLYKLIIIFLWLLDDDDD	-92700	✓
3	DTDDYYDTYTYYYFLLRKKKK	-92500	✓
4	EDDDWIMFWMTHGWTKRKRRR	-92300	X
5	EEEEFYIYETYWHYFYFKRRK	-92300	X
6	RRRKMTFWWWTFLVEEFMEEE	-91700	X
7	RRKYLLPWYIILYLAYWDDDD	-91600	✓
8	RWWCYWRPWYWWWRPWYPWWY	-91500	✓
9	KRKKKFWQWFQWMMEIWFEEE	-91400	✓
10	KRKKKTLNQWFLMLWDIDDEE	-90700	✓
11	YDDDMEWTYFYMWTTKRKKYK	-90500	✓
12	WDDDDILLLNVLYWPKRKKKL	-90200	✓
13	PWLYWWWPFPWPPIFPWPPPP	-90000	✓
14	LLPWFTPPPWWWLWWPPWGPP	-88800	✓
15	RRRKYMTKWQTEWWFEFEEEE	-88700	X
16	RRRKLKFWWTTLWEEQIEETE	-88500	X
17	RRKRTMLLAPLWPYWDSDDDD	-87800	✓
18	KRRKTWMYFYWWQYYFYWMEE	-87000	X
19	LDDDDWGLLCYLYFWCTRKKK	-86000	✓
20	DDDMDTTLTFTCYYFYRKRRK	-85900	✓
21	RKKKRLLLLKNLHLDLDDDDL	-85400	X
22	DDDLLLHWPLWLLTFWKKKRR	-85000	X
23	RRRRRILYYWRLYIYDLWPDD	-85000	X
24	KKKYKWYWKWYIGWFIWPDDD	-84700	X
25	TWEEEEYDWYTQWMNKKKKYK	-84700	✓
26	KRRRYLPWYILWLRLTWDWDD	-84700	✓
27	RRRKMTFWWWTWEIEETYWEE	-84600	X
28	DDDDDYGLYVILPIFGRKRKK	-84500	✓
29	DDDVDAVFYLWLGWGKRRKKR	-84000	X
30	KKKRYLLIQWLILQWIEQDED	-84000	✓
31	DDDIYYPPPYPPYYFRRFKKR	-83700	X
32	RKKRLGYLFPFWIVVDGDDDD	-83600	✓
33	DDLDLDWWGGTPWYPLKKRKR	-83500	✓
34	DLHYDDWFYLLLWRFRRFRRN	-83400	X
35	DEDMDMTLTYTWWFLITRKKK	-83200	✓
36	EEEELEQEQQWHELRWRRKKR	-81800	X
37	DEFEMLFFFFFWFFFFMWKKK	-81800	✓
38	EEDDLMNLMLMYYMTKRRKKL	-81600	X
39	DDDLYLWFYWWPLPPWPRKPK	-81300	✓
40	DDDDLALWWLGPLRIGPRRRK	-80900	✓
41	DDEMDTTLTFTWWFLIVRKKK	-80700	✓
42	KKTKRYMGLYWHPWDDDYDDD	-80700	X
43	EQEEEFFMFWQQFQWKKWKKR	-79900	X
44	YDDDDMHLTFRWMFLIPRKKK	-79600	✓
45	RRIRRIGCWWPWWPLWYVWDD	-79500	X
46	RMRKKKFKWMTKWTEETEEEE	-79400	X
47	DDDMQAFLTFTWWYLIMRKKK	-79100	X
48	RRRKTTTWYWTFWPEEFVDEF	-79000	✓
49	KMRKRFYWQHFYEWEQEEEWQ	-78500	X
50	EEEEWIHYTTYWYYFYIKPRK	-78300	X
51	RRRRRTTYYTYLPTAPPDDDD	-77900	✓
52	DDDDDLWFIFMPWMYTTTRRK	-77900	X
53	RRRRKKFKGCTLWAEEEEGEE	-77800	X
54	DDDDDYWYPYPWPPFYKGRIK	-77700	X
55	RTRFRRFKTQTYWIEETEEEE	-77600	X

Continued on next page

Index	Sequence	B_{22} ($\AA^3$)	PS
56	DDDTDTTLVYYYTTLLYKRKR	-77600	✓
57	RRRKKFFMHNHLTAEEMEEEW	-77400	X
58	IRRKKNFKWQTLWAEETEEEE	-77400	X
59	RKKKIPPWIPGYWADYWGDDD	-77300	✓
60	DEDDDSIPLTLGTYHWTKRRR	-77200	X
61	MRRKKKFKWQTLWIEFTEEEE	-77100	X
62	RRRKKRFMWTTTAFPETEEEE	-77000	X
63	DDDYLYYLDLYQLLLLRLRRR	-77000	✓
64	KKKYKSWLLLLYLLILWDDWD	-76100	✓
65	KKRKKMFKWQTLGAEETEEEE	-76000	X
66	WRKKNGWTTVLYYYYDWDDDD	-75200	X
67	KRRYRINLYYISMVDDGDLDD	-75000	X
68	KKNKKKLLLLLIYILEDDYDY	-74800	X
69	DDDDDFLNNINNWLNRNRRKN	-74400	X
70	DWDDGLLIPIWIIWIRIKWRR	-73800	✓
71	DDDPDYPIYIGMIPYIRRCRR	-73200	✓
72	DDDIDIILAPVWWIIWLPRKK	-73200	X
73	TWDDPWPYTWPYYWWPWPKKK	-72200	✓
74	LWDDHWWLLIYLLLFLLRRKW	-72100	✓
75	RRIKRYLLFNLLILTNVEDDD	-71400	✓

Sequences ranked by variance divergence index

The sequences below are ranked by their variance divergence index, Eq. (3). Larger values indicate that the contact-map variance is more spread across high-wavenumber modes. Table IV lists the top 10% of sequences by variance divergence index in the combined B_{22} dataset. Table V lists the bottom 10%, which serves as a control subset in our analysis. Each sequence's original table and index are noted for reference. The complete dataset can be obtained at `https://github.com/wmjac/heteropolymer-phase-separation`.

TABLE IV: Sequences with highest variance divergence index

Sequence	B_{22} ($\AA^3$) (σ^3)	Table
HPHPHPHPHPHPHPHPHPHPHP	-1000	T2 (Row 11)
HHHHPPHPHHHPPHHHHPPP	-1000	T2 (Row 25)
HHHHPPHPHHHPPHHHHPPP	-400	T1 (Row 26)
HHHPPPHHHPPHHHPPPHHH	-1000	T2 (Row 20)
HHHHPPPHHPHHPHHHHPPP	-1000	T2 (Row 24)
HHHHPPPHPHHPHHPHPHPH	-1000	T2 (Row 50)
HPHHPHHPPHHPPHHPHHPH	-400	T1 (Row 60)
HPHHPHPHPHHPHPHPHHPH	-400	T1 (Row 61)
PHPHPPHPPHPPHPPHPHPH	-1000	T2 (Row 1)
PHPHPPHPPHPPHPPHPPHH	-1000	T2 (Row 4)
HHHPHHPHHHPHHPHHPHHH	-1000	T2 (Row 59)
HHHHPPPHPHHPHHPHPHPH	-400	T1 (Row 52)
HHPHHPHPPHPHHHPPHPHH	-1000	T2 (Row 54)
HHPHHPHPPHPHHHPPHPHH	-400	T1 (Row 58)
HHHHPPPHHPHHPHHHHPPP	-400	T1 (Row 25)

TABLE V: Sequences with lowest variance divergence index

Sequence	B_{22} ($\AA^3$) (σ^3)	Table
PPHPPPHPPHPHHHHHPPPP	-400	T1 (Row 9)
PHPHHHHHHHHHHHHHPHPPH	-400	T1 (Row 36)
PHPHHHHHHHHHHHHHPHPPH	-1000	T2 (Row 35)
PPHPPPHPPHPHHHHHPPPP	-1000	T2 (Row 9)
PHPPPPPHHHHPHHPPPHPP	-1000	T2 (Row 6)
PHHPHPHHHHHHHPPPPHPH	-400	T1 (Row 28)
PHPPPPPHHHHPHHPPPHPP	-400	T1 (Row 6)
HPHPHHHHHHHHHPHPHHHP	-400	T1 (Row 34)
PPHHPHHHHHHHHHHHHHPH	-400	T1 (Row 42)
PPHHHHHHHHHHHHHHHHPP	-400	T1 (Row 43)
PPHHHPHPHPPPPPHPHPPH	-400	T1 (Row 8)
HPPPPHHPHHHHHHHPPHPH	-400	T1 (Row 30)
HPHHPPHHHHHHHHHHPHHH	-400	T1 (Row 73)
PHHPHPHHHHHHHPPPPHPH	-1000	T2 (Row 27)
PPHHPHHHHHHHHHHHHHPH	-1000	T2 (Row 41)
